# A Treatise on Headache and Neuralgia, Including Spinal Irritation, and a Disquisition on Normal and Morbid Sleep

**Published:** 1889-04

**Authors:** 


### Book Reviews.

---

A Treatise on Headache and Neuralgia, including Spinal Irritation and a Disquisition on Normal and Morbid Sleep. By J. Leonard Corning, M. A., M. D., 1888. E. B. Treat, New York.

In this book, Dr. Corning has added another to the series of excellent monographs which he has given to the profession. The succinct, pointed, easy style of the author is very pleasing.

The book is eminently practical, without any attempt at an exhaustive discussion of the various theories as to the causes of headaches and neuralgias, though the author evinces a thorough familiarity with the literature of the subjects.

Great stress is laid upon the benefit to be derived from carotid compression in that variety of headaches where there are marked suffusion of face and pulsation of the carotids, and the author has invented ingenuous instruments for mechanical and electro-compression of those vessels.

Antipyrine is also given a prominent place in the therapeutics of headaches.

The chapters on neuralgias and the local medication of nerves are excellent, and contain many valuable practical suggestions.

The treatment of spinal irritation by deep injections, so as to bring the medicament as nearly in contact with the cord as possible, is an innovation in therapeutics which is destined to revolutionize the treatment of that very troublesome class of cases. The technique of the operation is clearly described.

The chapters on normal and morbid sleep are entertaining and instructive, and the book, as a whole, is one which may be read with pleasure and profit by students and practitioners.

